# The Clinical Importance of IL-6, IL-8, and TNF-α in Patients with Ovarian Carcinoma and Benign Cystic Lesions

**DOI:** 10.3390/diagnostics11091625

**Published:** 2021-09-06

**Authors:** Weronika Pawlik, Jakub Pawlik, Mateusz Kozłowski, Karolina Łuczkowska, Sebastian Kwiatkowski, Ewa Kwiatkowska, Bogusław Machaliński, Aneta Cymbaluk-Płoska

**Affiliations:** 1 Department of Gynecological Surgery and Gynecological Oncology of Adults and Adolescents, Pomeranian Medical University in Szczecin, al. Powstańców Wielkopolskich 72, 70-111 Szczecin, Poland; jakubpawlik13@gmail.com (J.P.); mtkoozo@gmail.com (M.K.); aneta.cymbaluk@gmail.com (A.C.-P.); 2 Department of General Pathology, Pomeranian Medical University in Szczecin, al. Powstańców Wielkopolskich 72, 70-111 Szczecin, Poland; karolina.luczkowska@pum.edu.pl (K.Ł.); machalin@pum.edu.pl (B.M.); 3 Department of Obstetrics and Gynecology, Pomeranian Medical University in Szczecin, al. Powstańców Wielkopolskich 72, 70-111 Szczecin, Poland; kwiatkowskiseba@gmail.com; 4 Department of Nephrology, Transplantology and Internal Medicine, Pomeranian Medical University in Szczecin, al. Powstańców Wielkopolskich 72, 70-111 Szczecin, Poland; ewakwiat@gmail.com

**Keywords:** interleukin-6, interleukin-8, tumor necrosis factor alpha, ovarian cancer, benign cystic lesions, predicting factor, diagnostic markers

## Abstract

The exact pathogenesis and influence of various cytokines in patients with ovarian lesions remains unclear. Hence, this study aimed to investigate whether IL-6, IL-8, and TNF-α could be considered as new useful markers for diagnosis of ovarian cancer. 63 women diagnosed with ovarian cancer (OC) and 53 patients with benign ovarian cystic (BOC) lesions were included in this study. Serum levels of IL-6, IL-8, and TNF-α were measured using ELISA. Statistical comparisons were made using the Mann–Whitney U test and all correlations were evaluated by Spearman’s ranks. The serum IL-8 and TNF-α concentration measured in the OC Group was significantly higher than in the BOC Group (*p* < 0.05). The cutoff level of IL-8 and TNF-α in the serum was set at 4.09 ng/mL and 2.63 ng/mL, respectively, with the sensitivity and specificity of 70% and 96% for IL-8 and 85.7% and 79.3% for TNF-α (*p* < 0.0001). These results suggest that IL-8 and TNF-α are useful biomarkers for predicting the malignant character of lesions of the ovary. The present study highlighted the importance of measuring the cytokines such as IL-8 and TNF-α in patients with ovarian lesions in predicting the clinical outcome.

## 1. Introduction

Ovarian carcinoma is one of the most common types of neoplasms in women. Each year, more than 295 thousand new cases are diagnosed worldwide. It is one of the main causes of death in women, with nearly 185 thousand deaths per year [1]. Moreover, it has the highest case–to–mortality ratio among all gynecological malignancies [2].

Although the 5 year relative survival rate for women with localized ovarian cancer is quite high (92%) in many cases early-stage ovarian cancer might be asymptomatic, which poses a serious clinical issue and leads to late diagnosis mostly at regional or distant metastasis stage where the 5 year survival rate is 75% and 29%, respectively [2]. To date, the most efficient laboratory diagnostic tool for diagnosis of ovarian cancer is the combination of CA125 and HE4 called ROMA (Risk of Ovarian Malignancies Algorithm) [3]. However, its sensitivity (84%) and specificity (84%) could be improved [3]. Therefore, it is crucial to search for other markers useful in the diagnosis and prognosis of ovarian cancer at an early stage of its development to combine them with the ROMA algorithm to improve its diagnostic efficiency. 

Numerous studies have shown an association between the prolonged inflammation process of the ovarian surface epithelium and the increased risk of developing ovarian cancer in the future. Studies showed that cytokine signaling, including interleukins (IL) such as IL-6, IL-8, and TNF-α, plays an important role in regulating this process by promoting tumor initiation, growth, and cancer progression [4]. Levels of various cytokines have been shown to be increased in patients with ovarian cancer compared to nonmalignant lesions [5]. Cytokines can activate the growth of tumor cells by functioning as growth factors, controlling apoptosis, promoting metastasis and cell invasion, and amplifying tumor angiogenesis. They can also modulate the immune system to prevent it from destroying cancer cells [6].

Tumor Necrosis Factor alpha (TNF-α) is one of the main acute-response factors in the human body. Though it is mainly produced by macrophages, other cells (such as fibroblasts, neutrophils, smooth muscle cells, and neoplasm cells) can also be a source of TNF-α [7]. There are two types of TNF receptors: TNFR1 found on most human cells and TNFR2, which is found mostly on epithelial and hematological cells [8,9]. Though TNF-α is most known to induce apoptosis and regulate the inflammatory process to inhibit neoplasm cells, research shows that the presence of TNF-α in a tumor microenvironment might promote angiogenesis, migration, and cell invasion [10,11,12]. 

As a response of increased TNF-α and IL-1 serum levels, interleukin-6 can be excreted. It is one of the most important and multidirectional cytokines and can be produced by practically every stromal and immune cell [13]. IL-6 is a regulator of acute and chronic inflammatory reaction, promoter of expansion and activation of T-cells, and inducer of B-cell maturation, as well as apoptosis [13,14]. It acts through a group of Janus kinases and a protein family called signal transducers and activators of transcription [15,16]. 

Interleukin-8 is most associated with chemotaxis of neutrophils to the inflammation site, as well as the neovascularization process. IL-8 binds to two cell receptors both connected with the G-protein pathway: CXCR-1 and CXCR-2, which appear on the surface of various cells, such as human leukocytes, endothelial cells, and neoplasm cells [17,18]. It is presumed that IL-8 and its receptors may play a role in the development of malignancies [19]. A higher IL-8 expression was also investigated in other neoplasms. Its elevated serum concentration has also been reported, among others, in breast, prostate, and lung cancer [20,21,22,23]. An increased level of IL-8 was also present in the peritoneal fluid aspirated from patients with ovarian malignancies [24]. Fibroblasts obtained from patients with ovarian cancer show a higher expression of IL-8 compared to normal fibroblasts. Moreover, it might promote the migration of neoplastic cells [25].

Finding the connection between these three cytokines and ovarian cancer can enhance the efficiency of diagnosing malignant lesions, particularly differentiating them from benign ovarian pathologies (e.g., cysts) and healthy ovaries. The aim of this study was to investigate whether IL-6, IL-8, and TNF-α have sufficiently high sensitivity and specificity to be considered as new useful markers for diagnosis of ovarian cancer.

## 2. Materials and Methods

### 2.1. Participants

#### 2.1.1. Participation in the Study

A total of 116 female patients participated in the study. They were divided into two groups according to ovarian pathology. Sixty-three women with ovarian carcinoma (OC) were recruited among patients who were referred to the Department of Gynecological Surgery and Gynecological Oncology of Adults and Adolescents in Szczecin, Poland (hereinafter referred to as the Department) from January 2019 to November 2020. The presence of OC was confirmed according to the imaging studies and histopathological examination. For benign ovarian cysts (BOC), we recruited 53 patients among women who were referred to the Department from January 2019 to November 2020 due to the presence of a benign ovarian cystic lesion. Patients with malignant tumors were excluded from this study group. Study exclusion criteria were other types of gynecological neoplasms, comorbidity of nongynecological malignancies, acute inflammatory diseases, exacerbations of chronic inflammatory diseases, and autoimmune diseases during the study.

#### 2.1.2. Characteristics of the Study Group

Characteristics of patients with ovarian carcinoma and benign ovarian cystic lesions are demonstrated in Table 1 and Table 2 respectively.

The mean age of the OC patients was 62.3 ± 10.2 years and that of the BOC group was 58.01 ± 9.49 years. The OC patients were distributed according to their tumor stages as follows: 28 patients were in FIGO I-II, 30 patients in stage III-IV and 5 patients were unclassified. Out of the 63 patients with OC, 22 had a serous type of carcinoma. The nonserous ovarian cancers were distributed as follows: endometrial type-15 cases; mucous type-13 cases; and undifferentiated type-8 cases. For five cases, there were no histological data available. The mean BMI (Body Mass Index) of the OC patients was 26.9 ± 5.7 kg/m^2^, and in the BOC group, it was 23.3 ± 3.6 kg/m^2^.

### 2.2. Instruments

A specimen of the peripheral blood (5 mL) was obtained from all participants and the serum samples were separated. Concentrations of IL-6, IL-8, TNF-alpha were measured in peripheral blood plasma by the Luminex method based on color-coded superparamagnetic beads coated with analyte-specific antibodies (Luminex Corporation, Austin, TX, USA) using the commercial kit Luminex Human Discovery Assay (3-plex) (R&D Systems, Minneapolis, MN, USA). The procedure was performed according to the manufacturer’s protocol. In short, 50 μL of blank standards and samples were added to the 96-well plate and incubated with the microparticle cocktail for 2 h in the dark, at room temperature (RT), on a horizontal–orbital microplate shaker set at 750 rpm. After the incubation time has elapsed, the wells were washed three times with wash buffer 1× (100 μL/well). In the next step of the procedure, 50 µL of biotin–antibody cocktail was added to the plate and incubated for 1 h in the dark, at RT on the horizontal–orbital microplate shaker (750 rpm). In the last step of the procedure, streptavidin-PE (50 μL/well) was added to the plate and incubated for 30 min under the same conditions as the previous steps. Finally, the microspheres on the plate were washed three times, resuspended in wash buffer (100 μL/well), and read on the Luminex 200 analyzer (Luminex Corporation, Austin, TX, USA). The tested protein concentrations were calculated from a six-point standard curve.

The Complete Blood Count was performed using the Sysmex XN-2000 analyzer (Sysmex Europe GmbH, Norderstedt, Germany) during the hospital stay before the patient underwent a tumor excision. To perform comparative analysis of morphologic parameters, complete blood count with leukocyte differentiation (CBC+ 5-DIFF) was used.

### 2.3. Statistical Analysis

A Shapiro–Wilk test was used to check if the examined variables presented a normal distribution. The variables did not present a normal distribution, except for age in both study groups (W = 0.99157, *p* = 0.70221). Therefore, in the statistical analysis we have used nonparametric correlation (Spearman’s rank correlation coefficient) and relevance tests to evaluate the correlations between serum IL-6, IL-8, TNF-α levels, clinical characteristics, and selected blood count parameters. A nonparametric relevance U Mann–Whitney test was used (for independent samples) for statistical comparisons of IL-6, IL-8, TNF-α, CA125, HE4, WBC, NEU, LYM, and EOS serum levels between the group of OC and BOC patients. Differences between the groups were considered significant at *p* < 0.05. The critical cutoff values of IL-6, IL-8, and TNF-α were compared through a receiver operating characteristic (ROC) curve analysis.

## 3. Results

### 3.1. Serum Concentration of Studied Parameters

The serum IL-6 concentration in the OC Group (mean: 5.16 ng/mL) was higher than in the BOC Group (1.95 ng/mL). However, no significant difference was found between the IL-6 levels in groups OC and BOC (*p* = 0.22). The serum IL-8 concentration measured in the OC Group (mean: 17.28 ng/mL) was significantly higher than in the BOC Group (2.73 ng/mL, *p* < 0.05). The serum TNF-α concentration measured in OC Group (mean: 6.13 ng/mL) was significantly higher than in the BL Group (2.15 ng/mL, *p* < 0.05) (Table 3).

No significant difference was found between the IL-6, IL-8, and TNF-α serum levels and the histopathological type of cancer (serous vs. nonserous) (*p* = 0.31, *p* = 0.75, *p* = 0.82 respectively).

The serum NEU and LYM concentration measured in the OC Group (mean: 1.42 ng/mL and 3.54 ng/mL respectively) was significantly higher than in the BOC Group (mean: 1.35 ng/mL and 1.91 ng/mL respectively, *p* < 0.05). However, the concentration of serum EOS measured in the OC Group (mean: 0.06 ng/mL) was significantly lower than in the BOC Group (mean: 0.27 ng/mL, *p* < 0.05). No significant differences were found among groups regarding the concentrations of serum WBC (*p* = 0.83) (Table 4).

### 3.2. Correlations between Studied Parameters

Spearman rank correlation was performed to assess the association of the CA-125, HE-4, WBC, NEU, LYM, and EOS with serum IL-6, IL-8, and TNF-α. In the OC Group, there was a positive significant correlation between IL-6, IL-8, and CA125 (r = 0.418, *p* < 0.05; r = 0.314, *p* < 0.05). IL-8 levels also correlated with IL-6 and TNF-α (r = 0.511, *p* < 0.05; r = 0.553 *p* < 0.05, respectively). Significant positive correlations were found between serum IL-6 and serum HE-4 and TNF-α levels (r = 0.486, *p* < 0.05 and r = 0.461, *p* < 0.05). No significant correlation was found between WBC, NEU, LYM, EOS, and evaluated biomarkers. However, a statistically significant correlation was found between CA125 serum level and IL-6 and IL-8 serum levels in the BOC group (r = 0.404, *p* < 0.05; r = 0.566, *p* < 0.05 respectively). In this study, no significant relationship was observed between the FIGO stage and IL-6 (*p* = 0.8), IL-8 (*p* = 0.83) and TNF-α (*p* = 0.42). We have also evaluated the correlation between IL-6, IL-8, and TNF-α themselves. In both groups combined, we found that the strongest correlation was between IL-8 and TNF-α (r = 0.711, *p* < 0.05). Other correlations between the three markers were also statistically significant, but the correlation strength did not exceed r = 0.350. All the correlations are shown in Table 5.

### 3.3. Receiver Operating Characteristic (ROC) Curve for Using IL-6, IL-8, and TNF-α in Distinguishing between Ovarian Carcinoma and Benign Ovarian Cystic Lesion

**Figure 1:**
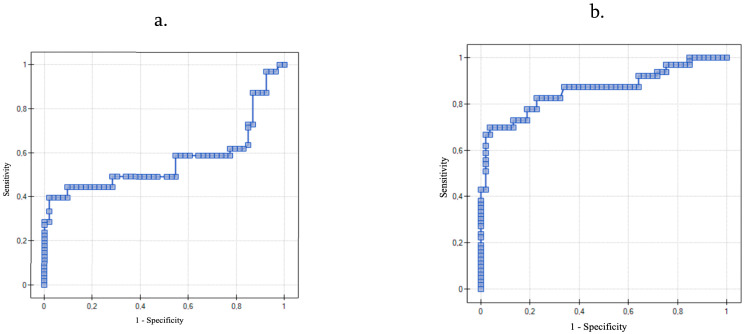

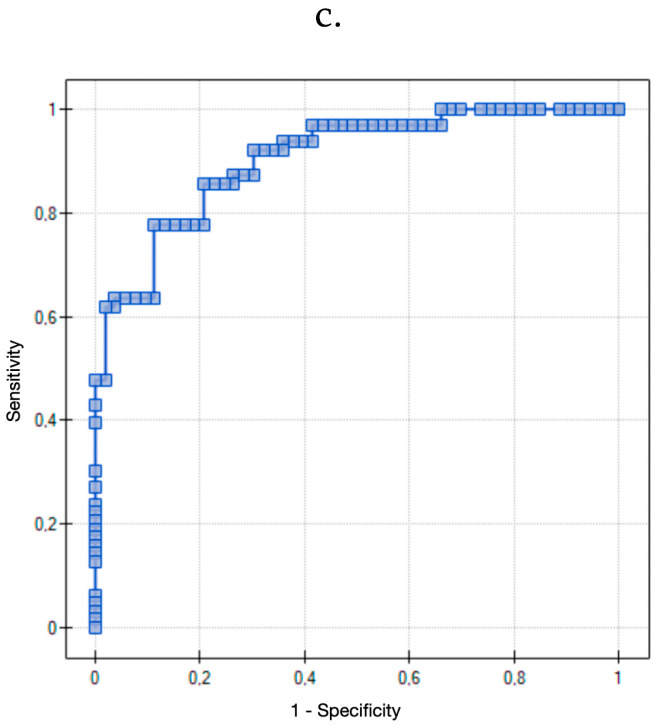
Receiver operating characteristic curve for using (**a**) IL-6 (**b**) IL-8, and (**c**) TNF-α in distinguishing between ovarian carcinoma and benign ovarian cystic lesions.

The cutoff values for the interleukin 6, interleukin 8, and TNF-α that were elevated in patients with ovarian carcinoma (OC Group) were calculated using ROC curve analysis. The analysis revealed that when the serum IL-6 concentration was 3.02 ng/mL or higher, the sensitivity and specificity were 40% and 98%, respectively (*p* = 0.22). When the serum IL-8 level was 4.09 ng/mL or higher, the sensitivity and specificity of a diagnosis of ovarian carcinoma were 70% and 96%, respectively (*p* < 0.0001); and when the serum TNF-α concentration was 2.63 ng/mL or higher, the sensitivity and specificity were 85.7% and 79.3% respectively (*p* < 0.0001) (Table 6). The ROC curves for IL-6, IL-8, and TNF-α are shown in Figure 1.

The sensitivity and specificity of IL-8 and TNF⍺ in distinguishing between malignant and benign ovarian lesions was calculated to be 78% and 82% for IL-8 and 84% and 81% for TNF⍺, respectively. Then an analysis of IL-8 and TNF⍺ as a combined parameter was performed. This calculation yielded an even higher sensitivity and specificity than using those parameters separately (88% and 82% respectively; *p* = 0.002). The results are presented in Table 7.

## 4. Discussion

Ovarian carcinoma is the most common of all gynecological malignancies, with high mortality [2]. Its prognosis is poor due to its highly metastatic nature. Therefore, research into the pathogenesis, detection, and treatment of this disease is still needed. To our knowledge, previous studies have rarely analyzed the clinical importance of serum levels of IL-6, IL-8, and TNF-α altogether in patients with ovarian carcinoma and ovarian benign cystic lesions. In our study, we found a positive correlation between the occurrence of ovarian cancer and the level of IL-8 and TNF-α in patient’s serum. Moreover, the data showed that the level of these biomarkers is associated with a widely known ovarian cancer biomarker-CA125.

As shown in this study, serum IL-6 levels were higher in the OC group than in the BOC group. However, the insufficient level of statistical significance does not allow us to apply this claim to the general population. It should be noted that past studies have also reported elevated levels of IL-6 in ovarian cancer patients [5,26,27]. As reported by Kampan et al. the use of CA125 in combination with IL-6 achieved higher predictive values compared to CA125 alone [5]. In the present study, we also demonstrated a statistically significant correlation between CA125 and IL-6 in ovarian cancer patients. However, we must point out that we observed a similar relationship in patients with benign ovarian cysts. In view of the proinflammatory nature of interleukin 6, this may indicate that the inflammatory process plays an important role in the pathogenesis of both ovarian cancer and benign ovarian cysts. Elevated IL-6 serum levels are also observed in endometriosis [28,29]. Increased CA125 serum levels have also been reported in its case [30]. Along with CA125, HE4 is an important marker in the diagnosis of ovarian cancer. Our study also reports the presence of correlation between IL-6 and HE4 in the OC group, which was not observed in the BOC group. The study by Han et al. also indicates the importance of IL-6 correlation with HE4 and suggests a novel biomarker panel: CA 125/HE4/E-CAD/IL-6 [31].

In this study, the concentration of IL-8 was significantly higher in the OC Group than in the BOC group. These findings are consistent with other research, proving its significant role in the inflammatory process in ovarian cancer compared to nonmalignant lesions [32,33]. Aune et al. reported results similar to ours: the serum level of Interleukin 8 in patients suffering from ovarian carcinoma was significantly higher than in those with benign ovarian lesions [25]. They also reported no difference in the concentration of IL-8 related to the FIGO stage or histological type. Zhang et al. have proved that IL-8 serum levels were elevated in patients with malignant ovarian tumors, compared to healthy participants [34]. On the other hand, they showed that patients with ovarian carcinoma stage III-IV had higher levels of IL-8 than patients in stage I-II, which suggests that the IL-8 serum level is related to the clinical stage or pathological type of the cancer. Our study shows that there is no statistically significant correlation between IL-6, IL-8, TNF-α and FIGO staging. Inconclusive results may be caused by the small size of the study group, especially considering patients with low clinical stage of the ovarian carcinoma. Therefore, further research to evaluate this topic is necessary.

Similar results to those demonstrated by IL-8 were presented by serum levels of TNF-α-its concentration was significantly higher in the OC Group compared to the BOC group. Several studies are consistent with our findings and confirm diagnostic relevance of TNF-α in distinguishing malignant ovarian neoplasm from benign masses [35,36,37]. As mentioned before, CA125 is well-known to be a helpful factor in discriminating between benign and malignant lesions.

In terms of the factor of prognosing the character of the lesion, in our evaluation of IL8, its higher serum concentration was correlated with higher CA125 level in both examined groups. This might mean that in the case of ovarian lesions, both malignant and benign, IL-8 may promote the expression of CA125-directly or via other mediators. We found no such correlation between TNF-α and CA125 markers.

Moreover, to our knowledge, we are the first to determine the cutoff level of IL-8 and TNF-α in the serum that could be used in diagnosis of ovarian cancer. The receiver operating characteristic (ROC) is a graphic tool for measuring the strength of the test and its usefulness in differentiation between the occurrence of the disease and its absence [38]. We found that if the cutoff level of IL-8 in the serum is set at 4.09 ng/mL the sensitivity and specificity of the result is 70% and 96%. We also managed to set the cutoff level of TNF-α in the serum at 2.63 ng/mL with the sensitivity and specificity of the result being 85.7% and 79.3%. The AUC of TNF-α was even higher than that of IL-8, which underlines its clinical value.

A positive correlation between all three analyzed markers was found. The strongest correlation we found (with r = 0.711) was between IL-8 and TNF-α. Their synergistic action in neoplasm development as well as the mechanism of inducing one another’s production has been hypothesized by other authors [20,39]. Our findings might prove one or both hypotheses. Moreover, we have proved that using them both as a combined diagnostic parameter yields a high specificity and sensitivity in distinguishing ovarian cancer from benign lesions. There is a chance that the diagnostic value of this combined parameter might further be enhanced by applying additional variables or using it in specific subpopulations of patients. We believe that this issue is still worth investigating.

## 5. Conclusions

The presented study results, as well as reports from other authors indicate that IL-8 and TNF-α can be used as biomolecular markers to provide clues for distinguishing ovarian cancer from benign cystic lesions. Setting the cutoff level of IL-8 and TNF-α in the serum at 4.09 ng/mL and 2.63 ng/mL, respectively, allowed us to consider them as useful diagnostic tools. Even though our research did not find a correlation between the clinical stage of the cancer and the level of serum IL-6, IL-8, and TNF-α, there are other reports confirming that fact. Additional studies with a larger sample of patients are needed to confirm the role of IL-6 in ovarian cancer diagnostics as well as further evaluate the clinical importance of IL-8 and TNF-α in patients with ovarian carcinoma and benign cystic lesions. Therefore, it seems that determination of serum concentration of IL-8 and TNF-α, especially in combination with CA125 and HE4, could be an aid in the initial diagnosis to differentiate malignancies from benign lesions.

## Figures and Tables

**Table 1:** Demographic and clinicopathological characteristics for the enrolled ovarian cancer patients.

Characteristic	Value	Number of Patients
Age (years)	62.3 ± 10.2	63
Tumor type	Serous	22
	Non-serous	41
FIGO stage	I-II	28
	III-IV	30
	Not classified	5
BMI [kg/m^2^]	26.9 ± 5.7	63

**Table 2:** Demographic and clinicopathological characteristics for the enrolled benign ovarian cystic lesion patients.

Characteristic	Value	Number of Patients
Age (years)	58.0 ± 9.5	53
BMI [kg/m^2^]	23.3 ± 3.6	53

**Table 3:** Mean serum levels and ranges of IL-6, IL-8, TNF-α, Ca 125, and HE4 in Ovarian Carcinoma and Benign Ovarian Cystic Lesions groups.

Characteristic		OC	BOC	*p*-Value
IL-6 [ng/mL]	Mean level	5.16	1.95	0.22
	Range	0.76–74.40	0.57–3.37	
IL-8 [ng/mL]	Mean level	17.28	2.73	<0.00001
	Range	1.70–332.60	0.97–5.60	
TNF⍺ [ng/mL]	Mean level	6.13	2.15	<0.00001
	Range	1.89–76.00	1.21–3.24	
Ca 125 level [U/mL]	Mean level	347.50	18.55	0.00004
	Range	5.50–4648.00	6.20–38.20	
HE4 level [pmol/L]	Mean level	453.10	58.74	<0.00001
	Range	37.20–4474.00	41.10–97.60	

**Table 4:** Selected Blood Parameters in Ovarian Carcinoma and Benign Ovarian Cysts groups.

Characteristic		OC	BOC	*p*-Value
Leukocytes [10^9^/L]	Mean level	5.52	5.36	0.82952
	Range	2.01–14.15	2.36–8.75	
Neutrophils [10^9^/L]	Mean level	1.43	1.35	0.03582
	Range	0.59–2.64	0.01–2.51	
Lymphocytes [10^9^/L]	Mean level	3.54	1.91	<0.00001
	Range	0.68–11.36	0.95–3.73	
Eosinophils [10^9^/L]	Mean level	0.03	0.27	<0.00001
	Range	0.00–1.03	0.06–1.20	

**Table 5:** The correlations between IL-6, IL-8, and TNF-α, presented as the Spearman’s ranges‚ r’ correlation coefficient. *p* < 0.05 for all presented coefficients.

Variable	IL-6	IL-8	TNFα
IL-6	1.000	0.332	0.321
IL-8	0.332	1.000	0.711
TNF⍺	0.321	0.711	1.000

**Table 6:** The diagnostic values of IL-6, IL-8, and TNF-α for patients with ovarian malignancies.

Marker	AUC (95% CI)	Sensitivity [%]	Specificity [%]	*p*-Value	Cut-Off Value [ng/mL]
IL-6	0.57 (0.46–0.68)	39.7	98.1	0.22	3.02
IL-8	0.86 (0.79–0.93)	69.8	96.2	<0.0001	4.09
TNF⍺	0.91 (0.85–0.96)	85.7	79.3	<0.0001	2.63

**Table 7:** The diagnostic values of IL-8 and TNF-α separately and combined in distinguishing between malignant and benign ovarian lesions.

Variable	IL8	TNFα	IL8/TNFα Combined	*p*-Value
Sensitivity	78%	84%	88%	0.002
Specifity	82%	81%	82%	0.002

## Data Availability

The data presented in this study are available on request from corresponding author, W.P., upon reasonable request.

## Abbreviations

OC	ovarian carcinoma
BOC	benign ovarian cysts
IL	interleukin
TNF-α	tumor necrosis factor alpha
WBC	white blood cells
NEU	neutrophils
EOS	eosinophils
LYM	lymphocytes
ROC	receiver operating characteristic
BMI	body mass index
